# C2H2 Zinc Finger Proteins: Master Regulators of Abiotic Stress Responses in Plants

**DOI:** 10.3389/fpls.2020.00115

**Published:** 2020-02-20

**Authors:** Guoliang Han, Chaoxia Lu, Jianrong Guo, Ziqi Qiao, Na Sui, Nianwei Qiu, Baoshan Wang

**Affiliations:** ^1^Shandong Provincial Key Laboratory of Plant Stress, College of Life Sciences, Shandong Normal University, Jinan, China; ^2^College of Life Sciences, Qufu Normal University, Qufu, China

**Keywords:** abiotic stress, adaptation mechanism, C2H2 zinc finger proteins, plant, signaling pathways, stress response networks

## Abstract

Abiotic stresses such as drought and salinity are major environmental factors that limit crop yields. Unraveling the molecular mechanisms underlying abiotic stress resistance is crucial for improving crop performance and increasing productivity under adverse environmental conditions. Zinc finger proteins, comprising one of the largest transcription factor families, are known for their finger-like structure and their ability to bind Zn^2+^. Zinc finger proteins are categorized into nine subfamilies based on their conserved Cys and His motifs, including the Cys2/His2-type (C2H2), C3H, C3HC4, C2HC5, C4HC3, C2HC, C4, C6, and C8 subfamilies. Over the past two decades, much progress has been made in understanding the roles of C2H2 zinc finger proteins in plant growth, development, and stress signal transduction. In this review, we focus on recent progress in elucidating the structures, functions, and classifications of plant C2H2 zinc finger proteins and their roles in abiotic stress responses.

## Introduction

As environmental problems become more severe, abiotic stress factors such as high salinity, low temperature, and drought will increasingly limit plant growth and crop yields (Chen et al., 2018; Costa and Farrant, 2019). During the long process of evolution, plants have evolved protective mechanisms in response to stress involving morphological, physiological, and molecular adaptations (Meng et al., 2018; Bernstein, 2019; Kumari et al., 2019). When a plant is subjected to abiotic stress, a series of adaptive changes occur in the plant cells to maintain growth, including the upregulation or downregulation of various genes (Cui et al., 2018; Yang et al., 2018; Han et al., 2019b).

Regulatory proteins function in stress signal transduction by influencing the expression of downstream target genes (functional genes). These regulatory proteins include protein kinases [including mitogen activated protein kinases (MAPK), calcium dependent protein kinases (CDPK), receptor protein kinases, ribosomal protein kinases, and transcriptional regulatory protein kinases] (Tibbles and Woodgett, 1999; Wimalasekera and Scherer, 2018; Divya et al., 2019), protein phosphatases (Singh et al., 2010; Gong et al., 2018), transcription factors (TFs) (Han et al., 2014; Sun et al., 2018), and proteins involved in inorganic phosphate (Pi) turnover (Takahashi et al., 2001; Fabiańska et al., 2019). TFs bind to specific sequences in the promoters of their target genes, thereby regulating gene expression and affecting biological phenotypes (Han et al., 2019a). TFs are key regulatory components of abiotic stress signaling pathways (Schmidt et al., 2012; Castelán-Muñoz et al., 2019).

Zinc finger proteins form one of the largest TF families in plants and are categorized into subfamilies based on the order of the Cys and His residues in their secondary structures, such as Cys2/His2-type (C2H2), C3H, C3HC4, C2HC5, C4HC3, C2HC, C4, C6, and C8 (Miller et al., 1985; Klug, 2010; Han et al., 2014). Among these, C2H2-type zinc finger protein genes account for ~0.7% of *Arabidopsis thaliana* genes, 0.8% of yeast genes, and 3% of dipteran and mammalian genes (Böhm et al., 1997; Englbrecht et al., 2004; Ciftci-Yilmaz and Mittler, 2008).

The first C2H2-type zinc finger protein gene discovered in plants was *EPF1* from *Petunia*, encoding a protein with two typical C2H2 zinc finger motifs. Fourteen *EPF1*-related genes are present in the *Petunia* genome (Takatsuji et al., 1992). Many C2H2-type zinc finger protein genes have since been cloned and studied in model plants such as *Arabidopsis*, wheat (*Triticum aestivum*), soybean (*Glycine max*), and rice (*Oryza sativa*) (Gao et al., 2011; Sun et al., 2012; Zhang et al., 2014; Hong et al., 2016). These genes encode proteins that play many roles in plant growth, development, and biotic stress resistance (Feurtado et al., 2011; An et al., 2012; Zhou et al., 2013; Yang et al., 2014; Weng et al., 2015; Cao et al., 2016).

In the past two decades, increasing evidence has indicated that C2H2-type zinc finger proteins also play important roles in abiotic stress resistance in plants. In this review, we describe the structures, classifications, and roles of plant C2H2-type zinc proteins in regulating abiotic stress responses in plants.

### Structure and Domain of C2H2 Zinc Finger Protein

**Figure 1:**
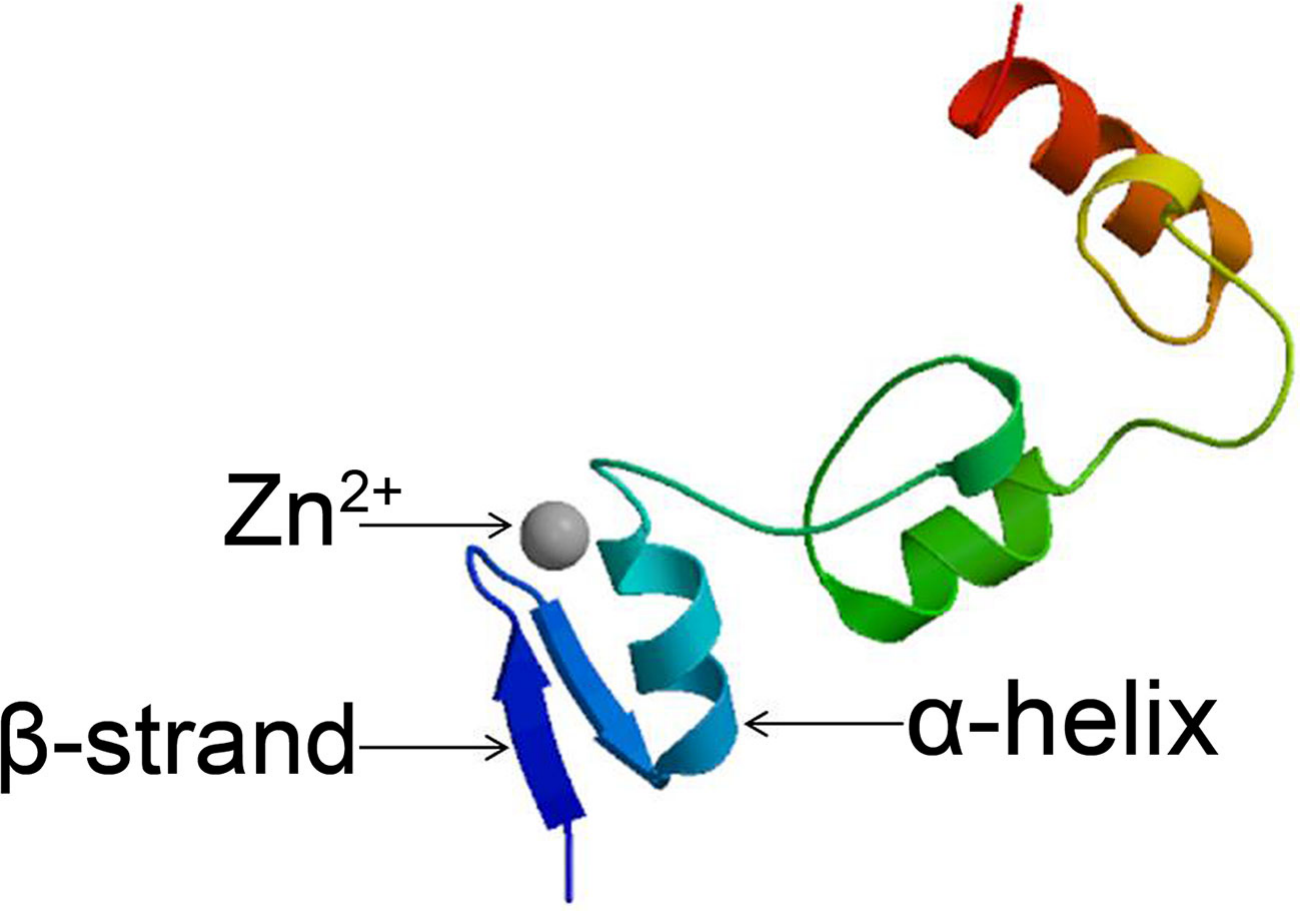
Structure of C2H2 zinc finger proteins. Structural model of the *Arabidopsis* C2H2 zinc finger protein STZ produced using the Protein Model Portal tool.

Zn^2+^ binds to several conserved amino acids in C2H2-type zinc finger proteins (generally Cys and His) to form a relatively independent region of the protein. Eukaryotic C2H2-type zinc finger proteins generally contain a specific conserved sequence consisting of 25 to 30 amino acids: C-X2~4-C-X3-P-X5-L-X2-H-X3-H (Shimeld, 2008; Finn et al., 2013). Two pairs of histidines at the C-terminus of the α-helix and the two cysteines at the end of the β-strand bind to Zn^2+^ to form a tetrahedral structure. Zn^2+^ is sandwiched between an α-helix and two antiparallel β-strands; the stability of the ββα structural system is maintained by the interlaced linkage provided by Zn^2+^ (**Figure 1**). The presence of Zn^2+^ ensures that the stability of the entire zinc finger structure and the normal helical structure are maintained (Wolfe et al., 2000; Alam et al., 2019).

Most C2H2 zinc finger proteins in plants contain the highly conserved sequence QALGGH in their zinc finger domains: such proteins are referred to as Q-type zinc finger proteins. Unlike proteins containing the QALGGH sequence, a class of C2H2-type zinc finger proteins do not contain any conserved motifs in the zinc finger region are known as C-type proteins (Agarwal et al., 2007; Liu et al., 2015b).

According to the number and distribution of zinc fingers, C2H2-type zinc finger proteins in plants are divided into four categories: (1) proteins containing one C2H2 zinc finger (single-C2H2); (2) proteins containing three C2H2 zinc fingers (triple-C2H2 [tC2H2]); (3) proteins containing more than three adjacent C2H2 zinc fingers (multiple-adjacent-C2H2 [maC2H2]); and (4) proteins containing several C2H2 zinc finger pairs that are widely separated (separated-paired-C2H2 [spC2H2]) (Wang et al., 2019).

## Transcriptional Regulation of C2H2 Zinc Finger Protein

### Transcriptional Activation

Although many C2H2 transcription factors show transcriptional activation activity, there is no general rule about particular domains that function as transcriptional activators among the various types of domains, such as proline-rich, serine-rich, glutamine-rich, and threonine-rich domains (De Pater et al., 1996; Kiełbowicz-Matuk, 2012; Tang et al., 2019). Many C2H2-type zinc finger proteins function as transcriptional activators during plant growth and abiotic stress, but the activation domain is poorly understood (Sakamoto et al., 2000; Xu et al., 2007; Zhou et al., 2011; Zhang et al., 2016a). Several stress-related C2H2-type zinc finger proteins contain a proline-rich region between the NLS and L-box motifs, which is considered to be the activation domain (Sakamoto et al., 2000).

### Transcriptional Inhibition

The associated amphiphilic repression (EAR) motif (^L^/_F_DLN ^L^/_F(x)_P) in plants is present in class II AP2/ERF proteins and in the C-termini of C2H2-type zinc finger proteins (TFIIIA class). The EAR motif reduces the underlying transcriptional level of the reporter gene, as well as the transcriptional activation activity of other TFs (Kazan, 2006). The conserved EAR motif in class II ERF TFs plays a key role in their inhibitory activity. For example, the C-terminal inhibitory domain of AtERF4 is DLDLNL; if a mutation occurs in this domain, the protein's inhibitory function is abolished (Ohta et al., 2001; Han et al., 2019a).

C2H2-type zinc finger proteins with EAR motifs play important roles in regulating abiotic-stress-related gene expression under conditions such as salt, drought, and low temperature (Han and Fu, 2019). The DLNL sequence (DLN-box) at the C-terminus of STZ, with trans-activation activity, functions in the transcriptional activation of *DREB1A* under salt stress (Sakamoto et al., 2004; Kazan, 2006; Zhang et al., 2016c; Li et al., 2018), while the DLN-box is not the only domain that determines the transcriptional inhibitory activity of C2H2 zinc finger proteins (Huang et al., 2009a).

## DNA/RNA/Protein Binding Diversity of C2H2 Zinc Finger Protein

### DNA-Binding Activity

Plant C2H2-type zinc finger proteins have unique structural features: the conserved, unique QALGGH (Q-type C2H2) motif and the long variable spacers between adjacent zinc finger domains (Klug and Schwabe, 1995; Takatsuji, 1999; Lyu and Cao, 2018). Both features are thought to be important for DNA-binding activity in plants (Kubo et al., 1998; Brayer et al., 2008). A mutation of any amino acid in QALGGH sequence except Q will cause the protein to completely lose its ability to bind to DNA, whereas a mutation in Q will greatly reduce its DNA binding ability (Kubo et al., 1998; Gourcilleau et al., 2011). Of course, characteristic region of a leucine-rich box (L-box) has also been identified to be related to protein interactions in these proteins (Sakamoto et al., 2000; Ciftci-Yilmaz et al., 2007). Bioinformatics tools such ZF Models (zinc finger specificity prediction based on the random forest model) (Gupta et al., 2014), ZifRC (zinc finger recognition code) (Najafabadi et al., 2015), and B1H screening of the C2H2-ZF domain (Persikov et al., 2015) have also been used for binding sequence prediction based on the C2H2-type zinc finger motif.

### RNA-Binding Activity

In addition to binding to DNA, C2H2-type zinc finger proteins can recognize RNA. Zinc fingers comprehensively recognize specific bases and specific folding backbones in diverse RNA structures (Brown, 2005; Lin and Lin, 2018). Contact with the phosphoric acid skeleton in RNA is essential for the recognition of this molecule. A phage display assay showed that the amino acids at positions −1 and +2 of the α-helix in C2H2 zinc finger proteins are important for RNA binding (Mcbryant et al., 1995).

### Protein-Binding Activity

Zinc finger proteins interact with other zinc finger proteins or other types of protein to regulate target gene expression (Brayer and Segal, 2008; Song et al., 2010). Zinc finger proteins interact with other zinc finger proteins to bind to other DNA sequences or to prevent binding to the corresponding DNA sequences, thereby regulating gene transcription and expression, the potential for mediating protein interactions is much larger compared to DNA binding (Shi and Berg, 1995; Mackay and Crossley, 1998; Gamsjaeger et al., 2007; Brayer et al., 2008; Yengo et al., 2018).

## Transcriptional Regulation of Target Genes by C2H2 Zinc Finger Proteins

*In vitro* experiments such as gel-shift and yeast one-hybrid assays are widely used to study the binding activity of plant C2H2-type zinc finger proteins to *cis*-elements in the promoter regions of their downstream genes (Sakamoto et al., 2004). Several abiotic-stress-related C2H2-type zinc finger proteins, such as AZF1, AZF2, AZF3, and ZAT10/STZ, specifically bind to the A(G/C)T repeat sequences in their target promoters (Takatsuji et al., 1994; Sakamoto et al., 2004; Kodaira et al., 2011). The DNA sequence A[AG/CT]CNAC (Sakai et al., 1995; Kubo et al., 1998; Zhou et al., 2011; Han et al., 2019a), the TGCTANNATTG element (Huang et al., 2009b), and TACAAT motifs (Shi et al., 2014) are also possible binding domains of C2H2 TFs in plants. Under abiotic stress conditions, C2H2 zinc finger proteins directly target downstream stress-related genes to activate or inhibit their expression.

C2H2 zinc finger proteins directly target downstream ion balance-related genes to improve salt resistance in plants (Sakamoto et al., 2004; Huang et al., 2007; Ma et al., 2016). C2H2 zinc finger proteins directly target key genes involved in the biosynthesis of osmotic adjustment substances to improve osmotic stress resistance in plants (Xu et al., 2008; Sun et al., 2010; Huang et al., 2012; Yu et al., 2014; Wang et al., 2016; Han et al., 2019b). C2H2 zinc finger proteins also directly target antioxidant genes associated with reactive oxygen species (ROS) scavenging under abiotic stress conditions (Rizhsky et al., 2004; Davletova et al., 2005a; Huang et al., 2009a; Sun et al., 2010; Chu et al., 2016; Liu et al., 2017; Li et al., 2018). C2H2 zinc finger proteins directly target C-repeat/DRE-binding factor genes (CBFs) to improve cold resistance in plants (Vogel et al., 2005). These proteins enhance salt tolerance by interacting with the miRNA-transport-related proteins (Ciftci-Yilmaz et al., 2007). Finally, C2H2 zinc finger proteins directly target downstream genes involved in hormone signal transduction (Kodaira et al., 2011; Kim et al., 2016; Yin et al., 2017).

## Roles of C2H2 Zinc Finger Proteins in Abiotic Stress

### Roles in Salt Stress

Salt stress is a serious problem that limits crop production worldwide (Munns and Tester, 2008; Chen et al., 2018). Many C2H2-type TFs act as transcriptional activators or repressors to regulate plant responses to salt stress (Han et al., 2014). Salt stress generates ionic, osmotic, and oxidative stress to plants (Song et al., 2011; Liu et al., 2018). C2H2-type zinc finger proteins participate in salt tolerance by influencing salt-regulated genes.

One way that C2H2-type zinc finger proteins can improve plant salt tolerance is by helping maintain ionic balance. STZ, the first salt-tolerance-related zinc finger protein identified, eliminates the Na^+^ sensitivity of calcineurin-deficient yeast. STZ appears to be partially dependent on ENA1/PMR2, a P-type ATPase required for Li^+^ and Na^+^ efflux in yeast. STZ might enhance salt tolerance in plants by regulating the expression of downstream ion-balance-related genes (Lippuner et al., 1996). *Arabidopsis* AZF1 and AZF3 respond rapidly to salt stress and may improve salt tolerance by regulating downstream *ENA1*-like genes (Sakamoto et al., 2000; Sakamoto et al., 2004). In rice, the *ZFP182* promoter appears to respond only to monovalent cations such as Na^+^. Therefore, ZFP182 might regulate downstream ion-transport-related genes, such as Na^+^ transporter genes, in the salt regulatory signaling pathway (Huang et al., 2007). Finally, *TaZNF* improves salt tolerance in wheat by increasing the excretion of Na^+^ and reducing stomatal aperture (Ma et al., 2016).

C2H2-type zinc finger proteins also improve salt tolerance by increasing the concentrations of osmotic adjustment substances. ZFP252 (Xu et al., 2008) and ZFP179 (Sun et al., 2010) improve salt tolerance in rice by increasing the contents of free proline and soluble sugars and upregulating various genes involved in the biosynthesis of osmotic substances, such as *OsDREB2A*, *OsP5CS, OsProT*, and *OsLea3*. In addition, IbZFP1 in sweet potato (*I. batatas* (L.) Lam.) (Wang et al., 2016), ZFP3 (Zhang et al., 2016a), and AtSIZ1 (Han et al., 2019b) in *Arabidopsis* enhance plant salt tolerance by increasing proline biosynthesis and accumulation.

C2H2-type zinc finger proteins also improve plant salt tolerance by increasing the ability to scavenge ROS. *Arabidopsis* plants constitutively expressing *ZAT10* show enhanced expression of the ROS scavenging enzymes ascorbate peroxidase1 (APX1), APX2, and Fe-superoxide dismutase1 (FSD1) (Mittler et al., 2006). Transgenic tomato and *Arabidopsis* plants overexpressing *SlZF3* show significantly increased levels of ascorbic acid. Bimolecular fluorescence complementation (BiFC) and Co-Immunoprecipitation (Co-IP) experiments show that SlZF3 directly binds CSN5B, a key component of the COP9 signalosome; CSN5B can promote the degradation of AsA in *Arabidopsis* by binding to VTC1, so the interaction of SlZF3 and CSN5B inhibited the binding of CSN5B to VTC1, which contributes to AsA biosynthesis (Li et al., 2018).

Many C2H2-type zinc finger proteins enhance plant salt tolerance through abscisic acid (ABA)-mediated signaling pathways. In addition to high salt, *AZF2* (Sakamoto et al., 2000; Sakamoto et al., 2004) and *StZFP1* (Tian et al., 2010) respond rapidly to ABA, suggesting that they improve salt tolerance *via* an ABA-dependent pathway. In *Thellungiella*, *ThZF1*, like AZF2, regulates downstream gene expression under salt stress (Xu et al., 2007). Transgenic *Arabidopsis* plants harboring *GhDi19-1* and *GhDi19-2* from cotton were more sensitive to salt and ABA than the wild type, suggesting that these genes are involved in plant adaptation to salt stress *via* the ABA signaling pathway (Li et al., 2010).

The mitogen-activated protein kinase (MAPK) cascade is involved in various biotic and abiotic stress responses. *ZAT6*-overexpressing lines in *Arabidopsis* show improved seed germination under salt stress *via* ZAT6's effects on the MAPK cascade. ZAT6 interacts with the stress-responsive MAPK MPK6, and the phosphorylation of ZAT6 by MPK6 is required to improve salt tolerance during seed germination (Liu et al., 2013b). *OsZFP213*-overexpressing rice lines are more salt tolerant than wild-type and RNAi lines. Yeast two-hybrid, pull-down, and BiFC experiments demonstrate that OsZFP213 interacts with OsMAPK3 to regulate salt tolerance (Zhang et al., 2018).

C2H2-type zinc finger proteins can improve plant salt tolerance *via* several mechanisms simultaneously, such as promoting ionic balance, scavenging ROS, and increasing the levels of osmotic adjustment substances. *GsZFP1* from soybean enhances salt tolerance in transgenic alfalfa by maintaining ionic balance, removing peroxides, and increasing the levels of osmotic adjustment substances (Tang et al., 2013). *TaZFP1* overexpression lines show improved salt tolerance due to increased photosynthesis, osmolyte accumulation, and ROS scavenging. RNA-seq analysis show that TaZFP1 regulates photosynthesis, osmolyte metabolism, and ROS-homeostasis-related genes involved in salt stress (Sun et al., 2019a). AtRZFP enhances salt tolerance by increasing ROS scavenging and maintaining Na^+^ and K^+^ homeostasis (Zang et al., 2016).

C2H2-type zinc finger proteins can also improve salt tolerance *via* other mechanisms. ZAT7 may enhance salt tolerance by interacting with the miRNA-transport-related proteins WRKY70 and HASTY (Ciftci-Yilmaz et al., 2007). A DST loss-of-function rice mutant shows enhanced salt tolerance due to increased stomatal closure and reduced stomatal density (Huang et al., 2009b). ZjZFN1 from *Zoysia japonica* increases salt tolerance in transgenic *Arabidopsis*. RNA-seq analysis of these plants suggests that ZjZFN1 regulates phenylalanine metabolism, α-linolenic acid metabolism, and phenylpropanoid biosynthesis during salt stress (Teng et al., 2018).

### Roles in Osmotic Stress

Osmotic stress is induced by many abiotic stresses such as salinity, cold, and drought stress (Bashir et al., 2019). Osmotic stress causes physiological drought, ion imbalance, oxidative damage, and growth inhibition in plants (Yamaguchi-Shinozaki and Shinozaki, 2006). The ability of plants to resist osmotic stress is related to their ability to alter osmotic pressure and increase the biosynthesis of osmotic regulators (Zhu, 2016).

The C2H2 zinc finger proteins ZAT12 (Davletova et al., 2005b) and ZAT10 (Mittler et al., 2006) are involved in regulating the osmotic stress pathway in *Arabidopsis*. Both *ZAT10*-overexpressing lines and *zat10* mutant showed enhanced tolerance to osmotic stress (Mittler et al., 2006). ZAT10 is phosphorylated by MPKs that are thought to function in abiotic stress tolerance (Nguyen et al., 2012). ZAT10 is a positive regulator of osmotic stress responses that is regulated by MAP kinases in *Arabidopsis* (Nguyen et al., 2016). In rice, *RZF71* was strongly induced by 20% PEG6000 treatment, suggesting that RZF71 plays an important role in the response to osmotic stress (Guo et al., 2007). Moreover, six C2H2-type zinc finger proteins were reported to be involved in osmotic stress responses in poplar (Gourcilleau et al., 2011). Finally, heterologously expressing *GmZAT4* from soybean enhanced the tolerance of *Arabidopsis* to 20% PEG treatment. Expression analysis of marker genes indicated that GmZAT4 enhances osmotic stress tolerance *via* an ABA pathway in both soybean and *Arabidopsis* (Sun et al., 2019b).

### Roles in Cold Stress

Cold snap often cause serious damage to plants and can kill plants when severe (Liu et al., 2016; Wang et al., 2017). A comprehensive understanding of the mechanism underlying cold damage in plants would help resolve the problem of cold stress injury (Yang et al., 2013; Cheng et al., 2014; Sui, 2015; Pareek et al., 2017).

C2H2 zinc finger proteins enhance cold resistance by directly regulating downstream cold-related genes in plants. For instance, ZAT12 regulates cold acclimation by controlling the expression of 15 cold-suppressed genes and 9 cold-inducible genes. ZAT12 also downregulates the expression of CBF genes, suggesting it plays a negative role in plant adaptation to cold stress (Vogel et al., 2005). Transgenic *Arabidopsis* and tobacco (*Nicotiana tabacum*) plants overexpressing *SCOF-1* show increased expression of COR (cold-regulated) genes and enhanced cold tolerance. The SCOF-1 transgenic plants recover from chilling stress more rapidly than the control, and the T2 generation of SCOF-1 transgenic *Arabidopsis* still expresses cold regulatory genes, such as *COR15a*, *COR47*, and *RD29B*, at higher levels than the wild type at normal growth temperatures. SCOF-1 does not directly interact with the CTR/DRE, ABRE, or *cis*-acting elements in the promoter regions of COR genes *in vitro*, but yeast two-hybrid experiments demonstrated that SCOF-1 interacts with SGBF-1. Interestingly, SCOF-1 significantly enhances the activity of *SGBF-1* bound to ABRE sequences *in vitro*, thereby promoting COR genes' expression and enhancing cold tolerance. The interaction of these two proteins is required for the participation of ABRE in the expression of cold-regulated genes to enhance cold tolerance. In addition, SCOF-1 increases cold tolerance in transgenic sweet potato (Kim et al., 2011) and transgenic potato (Kim et al., 2016). *SlCZFP1* enhances cold tolerance in transgenic *Arabidopsis* and rice by inducing the constitutive expression of COR or cold-responsive genes (Zhang et al., 2011). The cold-stress-related gene *COR6.6* was significantly upregulated in GmZF1 transgenic plants, suggesting that GmZF1 regulates cold stress resistance in transgenic *Arabidopsis* by binding to the *COR6.6* promoter region *(*Yu et al., 2014*)*. In banana*, the overexpression of MaC2H2-2* and *MaC2H2-3* significantly represses the transcription of *MaICE1*, a key component of the cold signaling pathway. Therefore, MaC2H2s might enhance cold resistance in banana by suppressing the transcription of *MaICE1(*Han and Fu, 2019*)*.

C2H2 zinc finger proteins can enhance plant cold resistance by increasing the levels of osmotic substances. *ZFP182 significantly* enhances cold tolerance in rice overexpression lines by increasing the expression of *OsP5CS* and *OsLEA3* and the accumulation of osmoprotectants (Huang et al., 2012). *GmZF1* improved cold resistance in transgenic *Arabidopsis* by increasing proline and soluble contents and reducing membrane lipid peroxidation under cold stress *(*Yu et al., 2014*)*.

Finally, several C2H2-type zinc finger proteins regulate low-temperature stress through the ABA signaling pathway. SCOF-1 enhances plant cold tolerance through ABA-dependent signaling pathways (Kim et al., 2001). *GmZF1* is highly induced by ABA treatment, suggesting that GmZF1 might participate in an ABA-dependent signaling pathway *(*Yu et al., 2014*)*.

### Roles in Drought Stress

Drought is an important factor limiting plant growth worldwide, inducing adverse reactions such as osmotic imbalance, membrane system damage, and decreased respiratory and photosynthetic rates. Drought not only hinders plant growth and metabolism at various stages but also affects crop quality and yields (Bartels and Sunkar, 2005; Liu et al., 2015a; Guo et al., 2018).

C2H2 zinc finger proteins enhance plant drought resistance by increasing the levels of osmotic adjustment substances. For example, rice plants overexpressing *ZFP252* have a significantly higher survival rate than wild-type and antisense-*ZFP252* plants under drought stress. The overexpression lines exhibit enhanced expression of stress-related genes, such as *Oslea3*, *OsP5CS*, and *OsProT*, which contribute to the accumulation of osmotic substances (Xu et al., 2008). Overexpressing *OsMSR15* (Zhang et al., 2016c) and *ZFP3* in transgenic *Arabidopsis* (Zhang et al., 2016a) confers increase in drought tolerance by maintaining higher proline contents, reducing electrolyte leakage, and increasing stress-responsive gene expression.

C2H2 zinc finger proteins also enhance drought resistance by improving the ability to scavenge ROS. ZFP245 improves drought resistance in rice by enhancing the activities of the ROS scavenging enzymes superoxide dismutase (SOD) and peroxide (POD) and increasing resistance to H_2_O_2_ (Huang et al., 2009a). When *ZxZF* from the highly drought-tolerant plant *Zygophyllum xanthoxylum* is expressed under the control of the drought-inducible promoter *rd29A* in *Arabidopsis* and poplar, the resulting transgenic plants have significantly increased photosynthetic efficiency, ROS, and scavenging ability (Chu et al., 2016). *ZAT18* knock down mutant exhibits reduced drought tolerance, while the overexpression lines show higher leaf water content and higher antioxidant enzyme activity than wild-type plants under drought stress (Yin et al., 2017).

C2H2 zinc finger proteins also enhance drought resistance *via* the ABA and other signaling pathways. Under dehydration stress, *AZF2* expression is lower in the *aba1* and *abi1* mutants than in the wild type, indicating that *AZF2* confers drought stress tolerance through an ABA-dependent pathway (Sakamoto et al., 2004). *GmZFP3* plays a negative role in plant tolerance to drought stress. Transgenic *Arabidopsis* plants of *GmZFP3* show enhanced expression of ABA-related marker genes, including *DREB2A*, *LHY1*, *MYB2*, *RCI3*, *PAD3*, *CCA1*, and *UGT71B6*, suggesting that GmZFP3 regulates the drought stress response through an ABA-dependent signaling pathway (Zhang et al., 2016b). Transgenic plants overexpressing *OsMSR15* show hypersensitivity to exogenous ABA. Transgenic plants also showed increased expressions of a number of stress-responsive genes, including RD29A and DREB1A under drought stress, which suggested that OsMSR15 may play important roles in response to drought stress both in ABA-dependent and -independent pathways (Zhang et al., 2016c). In the *ZAT18* overexpression lines, RNA-seq analysis revealed the enrichment of pathways, such as hormone metabolism, stress response, and signaling pathways; stress-related genes, such as *COR47*, *ERD7*, *LEA6*, *RAS1*, and hormone-signaling-transduction-related genes, such as *JAZ7* and *PYL5*, appear to be the downstream target genes of ZAT18 (Yin et al., 2017).

C2H2-type zinc finger proteins can also improve plant drought resistance through various mechanisms at the same time. Transformation with *CgZFP1* (Gao et al., 2012) and *DgZFP3* (Liu et al., 2013a) from chrysanthemum, *BcZAT12* from tomato (Rai et al., 2013), and *IbZFP1* from sweet potato (Wang et al., 2016) improve drought resistance in transgenic plants by increasing the levels of osmotic adjustment substances, improving ROS scavenging ability, and regulating downstream stress response genes. The *Arabidopsis azf2* mutant expressing the *Thellungiella halophila* gene *ThZF1 driven by the 35S promoter* displays a flowering time phenotype similar to that of the wild type under drought stress. ThZF1 and AZF2 can both activate the transcription *of AtEPSP* (Xu et al., 2007)*. GsZFP1 overexpression* enhances drought tolerance in transgenic alfalfa. The expression levels of stress-responsive marker genes, including *MtCOR47*, *MtRAB18*, *MtP5CS*, and *MtRD2*, were much higher in these plants than in the wild type under drought stress (Luo et al., 2012a).

### Roles in Oxidative Stress

Abiotic stress usually leads to the production of ROS. Low levels of ROS benefit plant growth and development by functioning as signals and might play other important roles as well (Choudhury et al., 2017), while the excessive accumulation of ROS causes secondary damage to plants. In the presence of excessive ROS, C2H2 zinc finger proteins are expressed at higher levels to help maintain stable ROS levels in plants (Gadjev et al., 2006).

The expression of antioxidase genes that are associated with ROS scavenging under abiotic stress can be regulated by C2H2 zinc finger proteins (Liu et al., 2017). Ascorbate peroxidase 1 (Apx1) is an important scavenger of H_2_O_2_ in plants (Rizhsky et al., 2004). The expression levels of TF genes *ZAT12* and *ZAT7* increase with increasing H_2_O_2_ levels in the loss-of-function mutant *apx1*. Plants lacking ZAT12 are more sensitive to high H_2_O_2_ contents than wild-type plants (Rizhsky et al., 2004; Davletova et al., 2005a). *APX1*, *APX2*, and *FeSOD1* are significantly upregulated in ZAT10 and ZAT12 transgenic lines, but are significantly downregulated in *zat10* loss-of-function mutants under high-light stress (Mittler et al., 2006). DST directly binds the promoter region of *peroxidase24 precursor* to regulate its expression and reduces the accumulation of H_2_O_2_ in plants (Huang et al., 2009b). SOD and POD activity is significantly enhanced in *ZFP245* and *ZFP179* overexpression rice plants, thereby increasing resistance to abiotic stress and ROS at the seedling stage (Huang et al., 2009a; Sun et al., 2010). Under water and oxidative stress, overexpressing *ZFP36* reduces the damage from ROS by increasing SOD and APX activity, whereas ZFP36-RNAi lines exhibit severe ROS damage due to reduced SOD and APX activity (Zhang et al., 2014).

C2H2-type zinc finger proteins participate in antioxidative stress responses through ABA and MAPK signaling pathways. *ZFP36* is significantly induced by ABA-triggered increases in H_2_O_2_ and OsMPK activity. ZFP36 also increases the expression levels of NADPH oxidase and MAPK genes in the ABA signaling pathway. These findings indicate that ZFP36 is involved in regulating the crosstalk among NADPH oxidase, H_2_O_2_, and MAPK in the ABA signaling pathway (Zhang et al., 2014).

### Roles in High-Light Stress

Plants are highly sensitive to strong light. The ability of plants to withstand strong light depends on the duration of light exposure and light quality (Yang et al., 2019). Overexpressing the C2H2-type zinc finger protein gene *RHL41*, which is identical to the *ZAT12* gene in *Arabidopsis*, significantly improved resistance to high-light conditions, as manifested by dramatic changes in plant morphology, such as increased production of thin-walled tissues of the barrier and increased anthocyanin and chlorophyll contents (Iida et al., 2000). The expression of *ZAT12* increases in response to light stress (Iida et al., 2000; Davletova et al., 2005b). Perhaps ZAT12 is part of the active oxygen scavenging signal transduction pathway, which responds to H_2_O_2_ produced during light stress (Davletova et al., 2005b). *ZAT10* expression is induced by light stress, and *ZAT10*-overexpressing transgenic lines showed enhanced tolerance to photo-inhibitory light stress (Mittler et al., 2006; Kilian et al., 2007). Under high-light stress, the levels of Apx1, Apx2, and iron superoxide dismutase 1 (FSD1) significantly increased in *ZAT10*-overexpressing transgenic plants, whereas suppressed expression was observed in *ZAT10* knockout plants, suggesting that the increased tolerance of *ZAT10*-overexpressing plants to high light results from the specific expression of ROS clearance-related genes (Miller et al., 2008). However, to date, few studies have focused on the roles of zinc finger proteins in plant responses to high-light stress; more work needs to be done in this area.

## Relationship of C2H2 Zinc Finger Protein With Hormone Response

As is well known, many plant hormones participate in physiological adaptations to abiotic stress, and many stress related plant hormones that play an important role in resistance to many stresses mediated by C2H2 type zinc finger proteins have been studied. Abscisic acid (ABA), salicylic acid (SA), jasmonic acid (JA), and ethylene (ET) are important plant hormones in pathogens and abiotic stresses signaling pathways (Shi et al., 2014; Zhang et al., 2014; Ma et al., 2016; Zang et al., 2016).

Typically, ABA is responsible for plant defense against abiotic stresses such as salinity, cold, osmotic, drought, active oxygen, and heat (Lata and Prasad, 2011; Ryu and Cho, 2015; Wang et al., 2019). Many studies found that the application of exogenous ABA can induce the expression of many stress related marker genes (Zhu, 2002; Fujita et al., 2013; Nakashima and Yamaguchi-Shinozaki, 2013; Tang et al., 2013). There are at least two ABA-mediated signal pathways existing in plants to respond to abiotic stresses: ABA-dependent and ABA-independent pathways; the two pathways also have some degree of crosstalk in response to abiotic stresses (Zhu, 2002; Shinozaki et al., 2003; Yamaguchi-Shinozaki and Shinozaki, 2005; Yamaguchi-Shinozaki and Shinozaki, 2006). C2H2 zinc finger proteins can confer abiotic stress tolerance by increasing the contents of abscisic acid (ABA), proline, soluble sugars or chlorophyll, and by reducing the water loss rate (Luo et al., 2012b; Wang et al., 2016). Zinc finger proteins are also involved in high temperature (Kim et al., 2015) and H^+^ (Fan et al., 2015) tolerance mediated by ABA signaling pathways. C2H2 zinc finger protein genes improve plant tolerance to stresses by ABA-dependent pathway (Zhang et al., 2016b) or ABA-dependent pathway (Huang et al., 2007), whereas some genes improve plant tolerance to stresses through both ABA-dependent and ABA-independent pathways (Sun et al., 2010). Abiotic stresses, as well as ABA signaling pathways, together with C2H2 zinc finger proteins constitute a complex network and there is many overlaps between different signaling pathways (Knight and Knight, 2001; Kiełbowicz-Matuk, 2012; Alam et al., 2019).

Salicylic acid (SA), jasmonic acid (JA), and ethylene (ET) play major roles in biotic stress responses, as their levels increase in response to pathogen infection and herbivorous insect attack (Bari and Jones, 2009; Gamalero and Glick, 2012; Wasternack and Hause, 2013). Of course, the stress response of hormones to stress is not completely strict. SA is a phenolic hormone that functions as an important signaling molecule in plant responses to biotic and abiotic stress. C2H2 TFs are involved in the early response to SA in *Carica papaya* (Jiang and Pan, 2012). AtZAT6 plays important roles in stress responses by activating the expression of SA-related genes in *Arabidopsis* (Shi et al., 2014). JAs are important class of lipid phytohormones. *ZPT2-3* expression is induced by mechanical wounding and abiotic stress, a process mediated by a JA-dependent, ET-independent pathway (Sugano et al., 2003). *AtZFP11*-overexpressing lines exhibited changes in the expression of a number of genes involved in JA and stress responses (Dinkins et al., 2012). ETHYLENE RESPONSE FACTOR1 (ERF1), a downstream component of the ET signaling pathway, is involved in plant resistance to several necrotrophic fungi (Berrocal-Lobo et al., 2002). High salt levels and drought stress significantly induced the expression of *ERF1* in *Arabidopsis*. ERF1 specifically regulates gene expression by integrating the JA, ET, and ABA signaling pathways and plays a positive regulatory role in plant resistance to salt, drought, and heat stress (Cheng et al., 2013).

## Regulating Network Between C2H2 Zinc Finger Protein and Abiotic Stress

It is now clear that C2H2-type zinc finger proteins play central roles in plant responses to high salt, cold, osmotic, drought, and oxidative stresses. Identifying abiotic stress sensors is crucial for understanding the molecular mechanisms underlying abiotic stress resistance in plants. For example, Glycosyl inositol phosphorylceramide (GIPC) was recently shown to be a salt receptor in plants (Jiang et al., 2019). REDUCED HYPEROSMOLALITY-INDUCED CALCIUM INCREASE 1 (OSCA1) is considered to be an osmotic stress sensor, but how it perceives osmotic stress is not clear (Yuan et al., 2014). Chilling-Tolerance Divergence 1 (COLD1), which regulates cold stress perception in rice, is another potential stress receptor (Ma et al., 2015; Shi and Yang, 2015). However, much more effort will be needed to identify abiotic stress sensors and to unravel the detailed roles of C2H2-type zinc finger proteins in abiotic stress resistance in plants.

**Figure 2:**
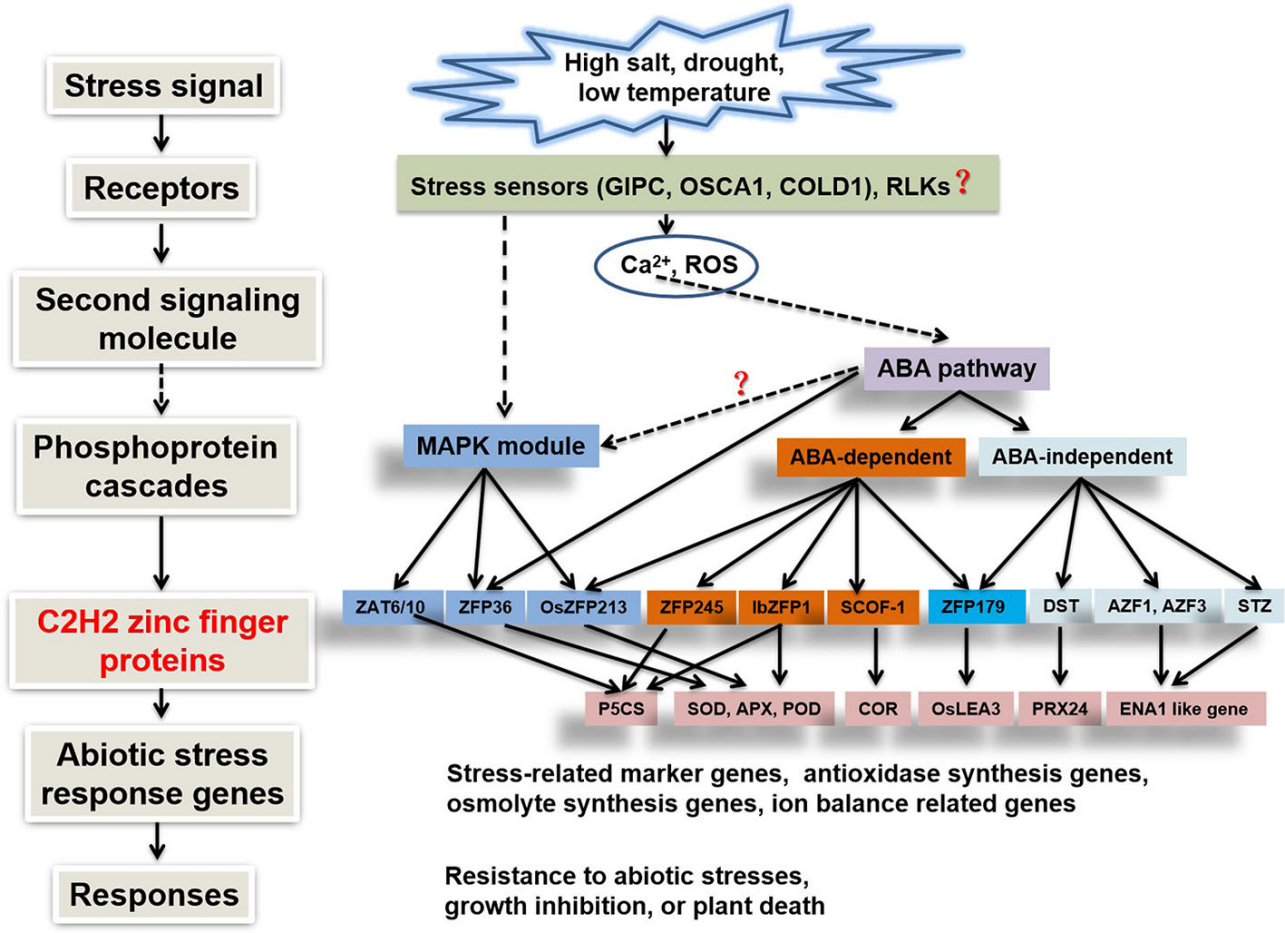
Signaling pathways of C2H2-type zinc finger protein genes involved in abiotic stress responses (Xiong et al., 2002; Wang et al., 2019).

After stress signals are perceived by the plant, second messengers (such as Ca^2+^ and ROS) are generated in the cytosol, where they mediate the phosphorylation of downstream proteins (by protein kinases such as MAPKs) as well as ABA-dependent or -independent pathways. Various *cis*-elements are present in the promoter regions of high salt-, drought-, and ABA-induced genes (Agarwal et al., 2006; Onishi et al., 2006). For example, C2H2-type zinc finger protein genes *AZF2, AtSIZ1*, and *STZ* contain *cis*-elements such as DRE (CCGAC), MYCRS (CANNTG), and MYBRS (RAACYR) elements in their promoter regions, conferring salt tolerance (Han et al., 2019). Protein kinases and ABA-dependent or -independent pathways (Zhang et al., 2014) directly or indirectly regulate C2H2 transcription factors, which in turn specifically activate or inhibit a group of target genes containing C2H2 zinc finger protein-binding sequences such as A [AG/CT]CNAC in their promoter regions, thereby enhancing plant stress tolerance (**Figure 2**).

Although abiotic stress responses are regulated by multiple pathways controlled by C2H2 zinc finger proteins, there is a certain degree of overlap among these signaling pathways and regulatory networks (Kiełbowicz-Matuk, 2012). The roles of plant C2H2-type zinc finger proteins in different abiotic stress responses and signaling pathways are summarized in **Supplementary Table 1**. As shown in the table, the same C2H2 zinc finger protein gene can be induced by multiple abiotic stresses at the same time. In addition, different C2H2 zinc finger proteins increase plant stress resistance through similar regulatory mechanisms, and different C2H2 zinc finger proteins improve plant stress resistance through similar ABA or protein kinase signaling pathways. Therefore, the stress response network of plants is very complex, and the many unknown proteins and related pathways must be further elucidated.

## Summary

C2H2-type zinc finger proteins play important roles in plant development and growth as well as abiotic stress resistance. In-depth research on these proteins is needed to better understand their functions and mechanisms.

The roles of C2H2 zinc finger proteins in plant stress-resistance mechanisms must be analyzed in more detail, such as how these transcription factors respond to various signaling molecules, how they regulate downstream gene expression, and how they interact with other transcription factors. Whether (and how) C2H2 zinc finger proteins mediate abiotic stress responses in plants by interacting with noncoding RNAs is currently unclear. The continuous development of biotechnology methods such as CRISPR-Cas should greatly facilitate the study of abiotic-stress-related C2H2-type zinc finger proteins in plants.

In addition to the model plant *Arabidopsis*, more studies should focus on the roles of C2H2-type zinc finger proteins in stress-resistant plants, such as halophytes (Yuan et al., 2015b) and xerophytes (Chu et al., 2016). During the course of evolution, stress-resistant plants have evolved various physiological and molecular mechanisms to adapt to stress (Sui et al., 2010; Sun et al., 2010; Feng et al., 2014). Halophytes can grow and finish their life cycles in saline soils with a salt concentration equivalent to ≥200mM NaCl (Santos et al., 2015; Yuan et al., 2015a; Zhou et al., 2016), and euhalophytes grow vigorously in sea water. C2H2 zinc finger proteins might be associated with these morphological features or play distinct roles in these plants. For instance, overexpressing the C2H2 zinc finger gene *ZjZFN1* from the halophyte *Z. japonica* improved salt tolerance in *Arabidopsis* by altering phenylalanine metabolism, α-linolenic acid metabolism, and phenylpropanoid biosynthesis (Teng et al., 2018). The expression of a homolog of the C2H2 zinc finger gene *AtSIZ1* from the halophyte *Limonium bicolor* improved salt tolerance in *Arabidopsis* by maintaining ionic homeostasis and osmotic balance (Han et al., 2019b).

These issues have been clarified in succession, which will be of great benefit to make full use of plant C2H2 zinc finger protein gene resources to improve crop varieties and increase plant stress resistance.

## Author Contributions

GH and BW conceived and designed this study. GH and BW wrote the manuscript. CL, JG, ZQ, NS, and NQ proposed related theories and assisted with the interpretation of some references. All authors have read, edited, and approved the current version of the manuscript.

## Funding

This work was supported by the NSFC (National Natural Science Research Foundation of China, project No. 31570251; 31600200; 31770288), the Shandong Province Key Research and Development Plan (2017CXGC0313; 2016GNC113012), the Natural Science Research Foundation of Shandong Province (ZR2014CZ002; ZR2017MC003; ZR2019MC065), the Higher Educational Science and Technology Program of Shandong Province (J15LE08; J17KA136), and the Open Fund of Shandong Provincial Key Laboratory of Plant Stress (KLPS2018-01).

## Conflict of Interest

The authors declare that the research was conducted in the absence of any commercial or financial relationships that could be construed as a potential conflict of interest.
