# Corrigendum: C2H2 Zinc Finger Proteins: Master Regulators of Abiotic Stress Responses in Plants

**DOI:** 10.3389/fpls.2020.00298

**Published:** 2020-03-18

**Authors:** Guoliang Han, Chaoxia Lu, Jianrong Guo, Ziqi Qiao, Na Sui, Nianwei Qiu, Baoshan Wang

**Affiliations:** ^1^Shandong Provincial Key Laboratory of Plant Stress, College of Life Sciences, Shandong Normal University, Jinan, China; ^2^College of Life Sciences, Qufu Normal University, Qufu, China

**Keywords:** abiotic stress, adaptation mechanism, C2H2 zinc finger proteins, plant, signaling pathways, stress response networks

In the original article, there was a mistake in Figure 1 as published. The labels of Figure 1 were inaccurate. We have re-checked the accuracy of Figure 1 using the Protein Model Portal tool shown in the published paper. The result showed that the tool has been disabled, and the Swiss-Mode tool was recommended. We have redrawn Figure 1 with the Swiss-Mode tool and ensured the correctness of the labels in Figure 1. The corrected Figure 1 appears below.

**Figure 1:**
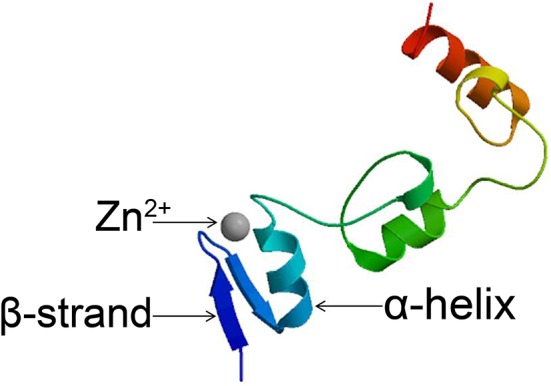
Structure of C2H2 zinc finger proteins. Structural model of the *Arabidopsis* C2H2 zinc finger protein STZ produced using the Swiss-Mode tool.

The authors apologize for this error and state that this does not change the scientific conclusions of the article in any way. The original article has been updated.

