# Supplementary material for: C2H2 Zinc Finger Proteins: Master Regulators of Abiotic Stress Responses in Plants
Source: Front Plant Sci. 2020 Feb 20;11:115. doi: 10.3389/fpls.2020.00115 (PMC7044346; doi:10.3389/fpls.2020.00115)
Supplement: Supplementary file 1 [file Table_1.docx]

**Supplementary Table 1 Roles of plant C2H2-type zinc finger proteins in different abiotic stress responses and signaling pathways**

| **Gene** | **Abiotic stress and signaling pathways** | | | | | | | | **Species** | | **References** |
| --- | --- | --- | --- | --- | --- | --- | --- | --- | --- | --- | --- |
|  | **Salt** | **Osmotic** | **Drought** | **Cold** | **ROS** | **Light** | **ABA pathways** | **MAPK module** | |  |  |
| *STZ* | **+** |  | **+** | **+** |  |  | **+** (dependent) |  | | *Arabidopsis thaliana* | ([Lippuner et al., 1996](#_ENREF_19);[Sakamoto et al., 2004](#_ENREF_33)) |
| *AZF1/2/3* | **+** |  | **+** | **+** |  |  | **+** (dependent) |  | | *Arabidopsis thaliana* | ([Sakamoto et al., 2000](#_ENREF_32);[Sakamoto et al., 2004](#_ENREF_33)) |
| *ZAT6* | **+** |  |  |  |  |  |  | **+** | | *Arabidopsis thaliana* | ([Liu et al., 2013b](#_ENREF_23)) |
| *Zat7* | **+** |  |  |  | **+** |  |  |  | | *Arabidopsis thaliana* | ([Rizhsky et al., 2004](#_ENREF_31);[Ciftci-Yilmaz et al., 2007](#_ENREF_2)) |
| *Zat10* | **+** | **+** | **+** |  | **+** | **+** |  | **+** | | *Arabidopsis thaliana* | ([Mittler et al., 2006](#_ENREF_27);[Miller et al., 2008](#_ENREF_26);[Nguyen et al., 2012](#_ENREF_29);[Nguyen et al., 2016](#_ENREF_28)) |
| *Zat12* |  | **+** |  | **+** | **+** | **+** |  |  | | *Arabidopsis thaliana* | ([Iida et al., 2000](#_ENREF_13);[Rizhsky et al., 2004](#_ENREF_31);[Vogel et al., 2005](#_ENREF_42)) |
| *ZAT18* |  |  | **+** |  |  |  | **+** |  | | *Arabidopsis thaliana* | ([Yin et al., 2017](#_ENREF_46)) |
| *ZFP3* | **+** |  | **+** |  |  |  |  |  | | *Arabidopsis thaliana* | ([Zhang et al., 2016a](#_ENREF_49)) |
| *AtSIZ1* | **+** |  |  |  |  |  | **+** |  | | *Arabidopsis thaliana* | ([Han et al., 2019](#_ENREF_5)) |
| *AtRZFP* | **+** |  |  |  |  |  |  |  | | *Arabidopsis thaliana* | ([Zang et al., 2016](#_ENREF_48)) |
| *ZFP182* | **+** |  | **+** | **+** |  |  | **+** (dependent) |  | | *Oryza sativa* | ([Huang et al., 2007](#_ENREF_11);[Huang et al., 2012](#_ENREF_9)) |
| *RZF71* | **+** | **+** | **+** |  |  |  |  |  | | *Oryza sativa* | ([Guo et al., 2007](#_ENREF_4);[Kim et al., 2007](#_ENREF_14)) |
| *RZF5* | **+** |  |  |  |  |  |  |  | | *Oryza sativa* | ([Guo et al., 2007](#_ENREF_4)) |
| *ZFP252* | **+** |  | **+** |  |  |  |  |  | | *Oryza sativa* | ([Xu et al., 2008](#_ENREF_44)) |
| *DST* | **+** |  | **+** |  | **+** |  | **+** (independent) |  | | *Oryza sativa* | ([Huang et al., 2009b](#_ENREF_12)) |
| *ZFP179* | **+** |  |  |  | **+** |  | **+** (dependent and independent) |  | | *Oryza sativa* | ([Sun et al., 2010](#_ENREF_36)) |
| *ZFP245* |  |  | **+** | **+** | **+** |  | **+** (independent) |  | | *Oryza sativa* | ([Huang et al., 2005](#_ENREF_10);[Huang et al., 2009a](#_ENREF_8)) |
| *OsZFP213* | **+** |  |  |  |  |  | **+** | **+** | | *Oryza sativa* | ([Zhang et al., 2018](#_ENREF_54)) |
| *ZFP36* |  |  |  | **+** |  |  | **+** | **+** | | *Oryza sativa* | ([Zhang et al., 2014](#_ENREF_51)) |
| *OsMSR15* |  |  | **+** |  |  |  |  |  | | *Oryza sativa* | ([Zhang et al., 2016c](#_ENREF_53)) |
| *TaZNF* | **+** |  |  |  |  |  | **+** |  | | *Triticum aestivum* | ([Ma et al., 2016](#_ENREF_25)) |
| *TaZFP1* | **+** |  |  |  |  |  | **+** |  | | *Triticum aestivum* | ([Sun et al., 2019a](#_ENREF_35)) |
| *ZmZF1* | **+** |  | **+** |  |  |  |  |  | | *Zea mays* | ([Huai et al., 2009](#_ENREF_7)) |
| *ThZF1* | **+** |  | **+** |  |  |  |  |  | | *Thellungiella halophila* | ([Xu et al., 2007](#_ENREF_45)) |
| *StZFP1* | **+** |  |  |  |  |  | **+** (independent) |  | | *Solanum tuberosum* | ([Tian et al., 2010](#_ENREF_40)) |
| *SICZFP1* |  |  |  | **+** |  |  |  |  | | Solanum lycopersicum | ([Zhang et al., 2011](#_ENREF_52)) |
| *SIZF3* | **+** |  |  |  |  |  |  |  | | *Solanum lycopersicum* | ([Li et al., 2018](#_ENREF_18)) |
| *GmZFP3* |  |  | **+** |  |  |  | **+** (dependent) |  | | *Glycine max* | ([Zhang et al., 2016b](#_ENREF_50)) |
| *SCOF1* |  |  |  | **+** |  |  | **+** (dependent) |  | | *Glycine max* | ([Kim et al., 2001](#_ENREF_15);[Kim et al., 2011](#_ENREF_16);[Kim et al., 2016](#_ENREF_17)) |
| *GmZF1* |  |  |  | **+** |  |  | **+** (dependent) |  | | *Glycine max* | ([Yu et al., 2014](#_ENREF_47)) |
| *GmZAT4* |  | **+** |  |  |  |  | **+** |  | | *Glycine max* | ([Sun et al., 2019b](#_ENREF_37)) |
| *GsZFP1* | **+** |  | **+** | **+** |  |  | **+** |  | | [*Glycine soja*](https://www.sciencedirect.com/topics/agricultural-and-biological-sciences/glycine-soja) | ([Luo et al., 2012](#_ENREF_24);[Tang et al., 2013](#_ENREF_38)) |
| *ZPT2-3* |  |  | **+** | **+** |  |  |  |  | | *Petunia hybrida* | ([van der Krol et al., 1999](#_ENREF_41);[Sugano et al., 2003](#_ENREF_34)) |
| *IbZFP1* | **+** |  | **+** |  |  |  | **+** (dependent) |  | | *Ipomoea batatas* | ([Wang et al., 2016](#_ENREF_43)) |
| *BcZAT12* |  |  | **+** |  |  |  |  |  | | *Solanum tuberosum* | ([Rai et al., 2013](#_ENREF_30)) |
| *CgZFP1* | **+** |  | **+** |  |  |  | **+** (independent) |  | | *Chrysanthemum grandiflorum* | ([Gao et al., 2012](#_ENREF_3)) |
| *DgZFP* | **+** |  |  |  |  |  | **+** (independent) |  | | Chrysanthemum | ([Liu et al., 2010](#_ENREF_21)) |
| *DgZFP3* |  |  | **+** |  |  |  |  |  | | *Chrysanthemum* | ([Liu et al., 2013a](#_ENREF_22)) |
| *PtrZPT2-1* | **+** |  | **+** | **+** |  |  | **+** (dependent) |  | | Poncirus trifoliata (L.) Raf. | ([Liu et al., 2017](#_ENREF_20)) |
| *ZxZF* |  |  | **+** |  |  |  |  |  | | *Zygophyllum xanthoxylum* | ([Chu et al., 2016](#_ENREF_1)) |
| *MaC2H2-1/2/3* |  |  |  | **+** |  |  |  |  | | Banana fruit (*Musa*) | ([Han and Fu, 2019](#_ENREF_6)) |
| *ZjZFN1* | **+** |  |  |  |  |  | **+** |  | | *Zoysia japonica* | ([Teng et al., 2018](#_ENREF_39)) |

**REFERENCES**

Chu, Y., Zhang, W., Wu, B., Huang, Q., Zhang, B., and Su, X. (2016). Overexpression of the novel Zygophyllum xanthoxylum C2H2-type zinc finger gene ZxZF improves drought tolerance in transgenic Arabidopsis and poplar. *Biologia* 71, 769-776.

Ciftci-Yilmaz, S., Morsy, M.R., Song, L., Coutu, A., Krizek, B.A., Lewis, M.W., Warren, D., Cushman, J., Connolly, E.L., and Mittler, R. (2007). The EAR-motif of the Cys2/His2-type zinc finger protein Zat7 plays a key role in the defense response of Arabidopsis to salinity stress. *Journal of Biological Chemistry* 282, 9260-9268.

Gao, H., Song, A., Zhu, X., Chen, F., Jiang, J., Chen, Y., Sun, Y., Shan, H., Gu, C., and Li, P. (2012). The heterologous expression in Arabidopsis of a chrysanthemum Cys2/His2 zinc finger protein gene confers salinity and drought tolerance. *Planta* 235, 979-993.

Guo, S.-Q., Huang, J., Jiang, Y., and Zhang, H. (2007). Cloning and characterization of RZF71 encoding a C2H2-type zinc finger protein from rice. *Yi chuan= Hereditas* 29, 607-613.

Han, G., Yuan, F., Guo, J., Zhang, Y., Sui, N., and Wang, B. (2019). AtSIZ1 improves salt tolerance by maintaining ionic homeostasis and osmotic balance in Arabidopsis. *Plant Science* 285, 55-67.

Han, Y.-C., and Fu, C.-C. (2019). Cold-inducible MaC2H2s are associated with cold stress response of banana fruit via regulating MaICE1. *Plant cell reports* 38, 673-680.

Huai, J., Zheng, J., and Wang, G. (2009). Overexpression of a new Cys 2/His 2 zinc finger protein ZmZF1 from maize confers salt and drought tolerance in transgenic Arabidopsis. *Plant Cell, Tissue and Organ Culture (PCTOC)* 99, 117-124.

Huang, J., Sun, S.-J., Xu, D.-Q., Yang, X., Bao, Y.-M., Wang, Z.-F., Tang, H.-J., and Zhang, H. (2009a). Increased tolerance of rice to cold, drought and oxidative stresses mediated by the overexpression of a gene that encodes the zinc finger protein ZFP245. *Biochemical and Biophysical Research Communications* 389, 556-561.

Huang, J., Sun, S., Xu, D., Lan, H., Sun, H., Wang, Z., Bao, Y., Wang, J., Tang, H., and Zhang, H. (2012). A TFIIIA-type zinc finger protein confers multiple abiotic stress tolerances in transgenic rice (Oryza sativa L.). *Plant molecular biology* 80, 337-350.

Huang, J., Wang, J.F., Wang, Q.H., and Zhang, H.S. (2005). Identification of a rice zinc finger protein whose expression is transiently induced by drought, cold but not by salinity and abscisic acid. *DNA Sequence* 16, 130-136.

Huang, J., Yang, X., Wang, M.-M., Tang, H.-J., Ding, L.-Y., Shen, Y., and Zhang, H.-S. (2007). A novel rice C2H2-type zinc finger protein lacking DLN-box/EAR-motif plays a role in salt tolerance. *Biochimica et Biophysica Acta (BBA)-Gene Structure and Expression* 1769, 220-227.

Huang, X.-Y., Chao, D.-Y., Gao, J.-P., Zhu, M.-Z., Shi, M., and Lin, H.-X. (2009b). A previously unknown zinc finger protein, DST, regulates drought and salt tolerance in rice via stomatal aperture control. *Genes & Development* 23, 1805-1817.

Iida, A., Kazuoka, T., Torikai, S., Kikuchi, H., and Oeda, K. (2000). A zinc finger protein RHL41 mediates the light acclimatization response in Arabidopsis. *The Plant Journal* 24, 191-203.

Kim, D.-W., Shibato, J., Agrawal, G.K., Fujihara, S., Iwahashi, H., Shim, I.-S., and Rakwal, R. (2007). Gene transcription in the leaves of rice undergoing salt-induced morphological changes (Oryza sativa L.). *Molecules & Cells (Springer Science & Business Media BV)* 24, 45-49.

Kim, J.C., Lee, S.H., Cheong, Y.H., Yoo, C.M., Lee, S.I., Chun, H.J., Yun, D.J., Hong, J.C., Lee, S.Y., and Lim, C.O. (2001). A novel cold‐inducible zinc finger protein from soybean, SCOF‐1, enhances cold tolerance in transgenic plants. *The Plant Journal* 25, 247-259.

Kim, Y.-H., Kim, M.D., Park, S.-C., Yang, K.-S., Jeong, J.C., Lee, H.-S., and Kwak, S.-S. (2011). SCOF-1-expressing transgenic sweetpotato plants show enhanced tolerance to low-temperature stress. *Plant Physiology and Biochemistry* 49, 1436-1441.

Kim, Y.H., Kim, M.D., Park, S.C., Jeong, J.C., Kwak, S.S., and Lee, H.S. (2016). Transgenic potato plants expressing the cold‐inducible transcription factor SCOF‐1 display enhanced tolerance to freezing stress. *Plant Breeding* 135, 513-518.

Li, Y., Chu, Z., Luo, J., Zhou, Y., Cai, Y., Lu, Y., Xia, J., Kuang, H., Ye, Z., and Ouyang, B. (2018). The C2H2 zinc‐finger protein Sl ZF 3 regulates AsA synthesis and salt tolerance by interacting with CSN 5B. *Plant biotechnology journal* 16, 1201-1213.

Lippuner, V., Cyert, M.S., and Gasser, C.S. (1996). Two classes of plant cDNA clones differentially complement yeast calcineurin mutants and increase salt tolerance of wild-type yeast. *Journal of Biological Chemistry* 271, 12859-12866.

Liu, D., Yang, L., Luo, M., Wu, Q., Liu, S., and Liu, Y. (2017). Molecular cloning and characterization of PtrZPT2-1, a ZPT2 family gene encoding a Cys2/His2-type zinc finger protein from trifoliate orange (Poncirus trifoliata (L.) Raf.) that enhances plant tolerance to multiple abiotic stresses. *Plant Science* 263, 66-78.

Liu, Q.-L., Xu, K.-D., Ma, N., Zeng, L., and Zhao, L.-J. (2010). Isolation and functional characterization of DgZFP: a gene encoding a Cys 2/His 2-type zinc finger protein in chrysanthemum. *Molecular biology reports* 37, 1137.

Liu, Q.-L., Xu, K.-D., Zhong, M., Pan, Y.-Z., Jiang, B.-B., Liu, G.-L., Jia, Y., and Zhang, H.-Q. (2013a). Overexpression of a novel chrysanthemum Cys2/His2-type zinc finger protein gene DgZFP3 confers drought tolerance in tobacco. *Biotechnology letters* 35, 1953-1959.

Liu, X.-M., Nguyen, X.C., Kim, K.E., Han, H.J., Yoo, J., Lee, K., Kim, M.C., Yun, D.-J., and Chung, W.S. (2013b). Phosphorylation of the zinc finger transcriptional regulator ZAT6 by MPK6 regulates Arabidopsis seed germination under salt and osmotic stress. *Biochemical and biophysical research communications* 430, 1054-1059.

Luo, X., Bai, X., Zhu, D., Li, Y., Ji, W., Cai, H., Wu, J., Liu, B., and Zhu, Y. (2012). GsZFP1, a new Cys2/His2-type zinc-finger protein, is a positive regulator of plant tolerance to cold and drought stress. *Planta* 235, 1141-1155.

Ma, X., Liang, W., Gu, P., and Huang, Z. (2016). Salt tolerance function of the novel C2H2-type zinc finger protein TaZNF in wheat. *Plant physiology and biochemistry* 106, 129-140.

Miller, G., Shulaev, V., and Mittler, R. (2008). Reactive oxygen signaling and abiotic stress. *Physiologia plantarum* 133, 481-489.

Mittler, R., Kim, Y., Song, L., Coutu, J., Coutu, A., Ciftci-Yilmaz, S., Lee, H., Stevenson, B., and Zhu, J.-K. (2006). Gain‐and loss‐of‐function mutations in Zat10 enhance the tolerance of plants to abiotic stress. *FEBS letters* 580, 6537-6542.

Nguyen, X.C., Kim, S.H., Hussain, S., An, J., Yoo, Y., Han, H.J., Yoo, J.S., Lim, C.O., Yun, D.-J., and Chung, W.S. (2016). A positive transcription factor in osmotic stress tolerance, ZAT10, is regulated by MAP kinases in Arabidopsis. *Journal of plant biology* 59, 55-61.

Nguyen, X.C., Kim, S.H., Lee, K., Kim, K.E., Liu, X.-M., Han, H.J., Hoang, M.H.T., Lee, S.-W., Hong, J.C., and Moon, Y.-H. (2012). Identification of a C 2 H 2-type zinc finger transcription factor (ZAT10) from Arabidopsis as a substrate of MAP kinase. *Plant cell reports* 31, 737-745.

Rai, A.C., Singh, M., and Shah, K. (2013). Engineering drought tolerant tomato plants over-expressing BcZAT12 gene encoding a C2H2 zinc finger transcription factor. *Phytochemistry* 85, 44-50.

Rizhsky, L., Davletova, S., Liang, H., and Mittler, R. (2004). The zinc finger protein Zat12 is required for cytosolic ascorbate peroxidase 1 expression during oxidative stress in Arabidopsis. *Journal of Biological Chemistry* 279, 11736-11743.

Sakamoto, H., Araki, T., Meshi, T., and Iwabuchi, M. (2000). Expression of a subset of the Arabidopsis Cys2/His2-type zinc-finger protein gene family under water stress. *Gene* 248, 23-32.

Sakamoto, H., Maruyama, K., Sakuma, Y., Meshi, T., Iwabuchi, M., Shinozaki, K., and Yamaguchi-Shinozaki, K. (2004). Arabidopsis Cys2/His2-type zinc-finger proteins function as transcription repressors under drought, cold, and high-salinity stress conditions. *Plant physiology* 136, 2734-2746.

Sugano, S., Kaminaka, H., Rybka, Z., Catala, R., Salinas, J., Matsui, K., Ohme‐Takagi, M., and Takatsuji, H. (2003). Stress‐responsive zinc finger gene ZPT2‐3 plays a role in drought tolerance in petunia. *The Plant Journal* 36, 830-841.

Sun, B., Zhao, Y., Shi, S., Yang, M., and Xiao, K. (2019a). TaZFP1, a C2H2 type-ZFP gene of T. aestivum, mediates salt stress tolerance of plants by modulating diverse stress-defensive physiological processes. *Plant Physiology and Biochemistry* 136, 127-142.

Sun, S.-J., Guo, S.-Q., Yang, X., Bao, Y.-M., Tang, H.-J., Sun, H., Huang, J., and Zhang, H.-S. (2010). Functional analysis of a novel Cys2/His2-type zinc finger protein involved in salt tolerance in rice. *Journal of experimental botany* 61, 2807-2818.

Sun, Z., Liu, R., Guo, B., Huang, K., Wang, L., Han, Y., Li, H., and Hou, S. (2019b). Ectopic expression of GmZAT4, a putative C2H2-type zinc finger protein, enhances PEG and NaCl stress tolerances in Arabidopsis thaliana. *3 Biotech* 9, 166.

Tang, L., Cai, H., Ji, W., Luo, X., Wang, Z., Wu, J., Wang, X., Cui, L., Wang, Y., and Zhu, Y. (2013). Overexpression of GsZFP1 enhances salt and drought tolerance in transgenic alfalfa (Medicago sativa L.). *Plant physiology and biochemistry* 71, 22-30.

Teng, K., Tan, P., Guo, W., Yue, Y., Fan, X., and Wu, J. (2018). Heterologous expression of a novel Zoysia japonica C2H2 zinc finger gene, ZjZFN1, improved salt tolerance in Arabidopsis. *Frontiers in plant science* 9, 1159.

Tian, Z.D., Zhang, Y., Liu, J., and Xie, C.H. (2010). Novel potato C2H2‐type zinc finger protein gene, StZFP1, which responds to biotic and abiotic stress, plays a role in salt tolerance. *Plant biology* 12, 689-697.

Van Der Krol, A.R., Van Poecke, R.M., Vorst, O.F., Voogd, C., Van Leeuwen, W., Borst-Vrensen, T.W., Takatsuji, H., and Van Der Plas, L.H. (1999). Developmental and wound-, cold-, desiccation-, ultraviolet-B-stress-induced modulations in the expression of the Petunia zinc finger transcription factor geneZPT2-2. *Plant Physiology* 121, 1153-1162.

Vogel, J.T., Zarka, D.G., Van Buskirk, H.A., Fowler, S.G., and Thomashow, M.F. (2005). Roles of the CBF2 and ZAT12 transcription factors in configuring the low temperature transcriptome of Arabidopsis. *The Plant Journal* 41, 195-211.

Wang, F., Tong, W., Zhu, H., Kong, W., Peng, R., Liu, Q., and Yao, Q. (2016). A novel Cys 2/His 2 zinc finger protein gene from sweetpotato, IbZFP1, is involved in salt and drought tolerance in transgenic Arabidopsis. *Planta* 243, 783-797.

Xu, D.-Q., Huang, J., Guo, S.-Q., Yang, X., Bao, Y.-M., Tang, H.-J., and Zhang, H.-S. (2008). Overexpression of a TFIIIA‐type zinc finger protein gene ZFP252 enhances drought and salt tolerance in rice (Oryza sativa L.). *FEBS letters* 582, 1037-1043.

Xu, S., Wang, X., and Chen, J. (2007). Zinc finger protein 1 (ThZF1) from salt cress (Thellungiella halophila) is a Cys-2/His-2-type transcription factor involved in drought and salt stress. *Plant cell reports* 26, 497-506.

Yin, M., Wang, Y., Zhang, L., Li, J., Quan, W., Yang, L., Wang, Q., and Chan, Z. (2017). The Arabidopsis Cys2/His2 zinc finger transcription factor ZAT18 is a positive regulator of plant tolerance to drought stress. *Journal of Experimental Botany* 68, 2991-3005.

Yu, G.-H., Jiang, L.-L., Ma, X.-F., Xu, Z.-S., Liu, M.-M., Shan, S.-G., and Cheng, X.-G. (2014). A soybean C2H2-type zinc finger gene GmZF1 enhanced cold tolerance in transgenic Arabidopsis. *PLoS One* 9, e109399.

Zang, D., Li, H., Xu, H., Zhang, W., Zhang, Y., Shi, X., and Wang, Y. (2016). An Arabidopsis zinc finger protein increases abiotic stress tolerance by regulating sodium and potassium homeostasis, reactive oxygen species scavenging and osmotic potential. *Frontiers in plant science* 7, 1272.

Zhang, A., Liu, D., Hua, C., Yan, A., Liu, B., Wu, M., Liu, Y., Huang, L., Ali, I., and Gan, Y. (2016a). The Arabidopsis Gene zinc finger protein 3 (ZFP3) is involved in salt stress and osmotic stress response. *PloS one* 11, e0168367.

Zhang, D., Tong, J., Xu, Z., Wei, P., Xu, L., Wan, Q., Huang, Y., He, X., Yang, J., and Shao, H. (2016b). Soybean C2H2-type zinc finger protein GmZFP3 with conserved QALGGH motif negatively regulates drought responses in transgenic Arabidopsis. *Frontiers in plant science* 7, 325.

Zhang, H., Liu, Y., Wen, F., Yao, D., Wang, L., Guo, J., Ni, L., Zhang, A., Tan, M., and Jiang, M. (2014). A novel rice C2H2-type zinc finger protein, ZFP36, is a key player involved in abscisic acid-induced antioxidant defence and oxidative stress tolerance in rice. *Journal of experimental botany* 65, 5795-5809.

Zhang, X., Guo, X., Lei, C., Cheng, Z., Lin, Q., Wang, J., Wu, F., Wang, J., and Wan, J. (2011). Overexpression of SlCZFP1, a novel TFIIIA-type zinc finger protein from tomato, confers enhanced cold tolerance in transgenic Arabidopsis and rice. *Plant molecular biology reporter* 29, 185-196.

Zhang, X., Zhang, B., Li, M.J., Yin, X.M., Huang, L.F., Cui, Y.C., Wang, M.L., and Xia, X. (2016c). OsMSR15 encoding a rice C2H2-type zinc finger protein confers enhanced drought tolerance in transgenic Arabidopsis. *Journal of Plant Biology* 59, 271-281.

Zhang, Z., Liu, H., Sun, C., Ma, Q., Bu, H., Chong, K., and Xu, Y. (2018). A C2H2 zinc-finger protein OsZFP213 interacts with OsMAPK3 to enhance salt tolerance in rice. *Journal of plant physiology* 229, 100-110.
